# Histological and micro‐computed tomography evaluations of newly formed bone after maxillary sinus augmentation using a xenograft with similar density and mineral content of bone: An experimental study in rabbits

**DOI:** 10.1002/cre2.146

**Published:** 2018-11-23

**Authors:** Takahisa Iida, Erick Ricardo Silva, Niklaus P. Lang, Karol Alí Apaza Alccayhuaman, Daniele Botticelli, Samuel P. Xavier

**Affiliations:** ^1^ Department of Oral Implantology Osaka Dental University Hirakata Japan; ^2^ Depto CTBMF e Periodontia FORP‐USP‐Faculty of Ribeirão Preto (SP) Ribeirão Preto Brazil; ^3^ Center for Dental Medicine University of Zurich Zurich Switzerland; ^4^ Department of Periodontology University of Bern Bern Switzerland; ^5^ ARDEC Academy Ariminum Odontologica Rimini Italy

**Keywords:** animal study, bone healing, collagen membrane, resorbable material, sinus floor elevation, xenograft

## Abstract

The objective of this study was to evaluate possible differences in the assessment of bone formation between histological and micro‐computed tomography (CT) analyses in maxillary sinuses augmented with a xenograft with similar density and mineral content of bone. A collagen membrane was placed subjacent the elevated sinus mucosa at the test sites of 18 rabbits, and the elevated spaces were filled with xenograft. The antrostomy was covered with collagen membranes, bilaterally. Six rabbits per group were sacrificed after 2, 4, and 8 weeks of healing. Biopsies were retrieved and scanned in a high‐resolution micro‐CT at two different gray thresholds. Histological assessments were subsequently performed. At the histological analyses, bone increased over time, from 7.5 ± 2.4% to 27.0 ± 5.3%, between 2 and 8 weeks of healing. The highest content of bone was found close to the sinus bone walls, whereas the middle regions contained lower amounts. At the micro‐CT analyses, discrepancies were found in bone content percentages compared with the histological analyses, especially after 2 weeks of healing and within the middle regions of the sinus, in which new bone was ~15–22% at the micro‐CT analyses and only 1.6% at the histological evaluation. The outcomes of a micro‐CT analysis performed in an early phase of healing may be altered when a resorbable bone substitute with similar density and mineral content of bone is applied.

## 1 INTRODUCTION

The healing after sinus floor elevation has been widely studied. It has been shown that new bone forms from the sinus floor and from the walls that have been exposed after the elevation of the Schneiderian membrane (Favero et al., 2016; Iida et al., 2017; Scala et al., 2010, 2012, 2016). A self‐containing space, such as that created after the elevation of the sinus mucosa, may yield great chances to be filled with newly formed bone. However, various factors will influence the healing outcome related to anatomical and physiological features, technical procedures, and biomaterials applied. Moreover, the tendency of the maxillary sinus to regain the lost space after sinus floor elevation has been documented as well (Asai, Shimizu, & Ooya, 2002; Caneva et al., 2017; De Santis et al., 2017; Scala et al., 2012, 2015; Xu, Shimizu, Asai, & Ooya, 2004). To counteract the physiological shrinkage of the elevated space, bone fillers of different characteristics have been applied in these procedures (Corbella, Taschieri, Weinstein, & Del Fabbro, 2016). Some of these materials are scarcely resorbed so that they will maintain most of the volume obtained. If the biomaterial used has osteoconductive properties, the bone growth may take advantages from the maintenance of the self‐contain effect. However, after healing, large regions of the elevated volume will still be occupied by bone substitutes, which will affect the characteristics of the bone (Caneva et al., 2017).

When a resorbable material is used to fill the elevated space, the self‐maintaining effect will be partly lost during healing so that portions of the elevated volume will also be lost (Caneva et al., 2017; De Santis et al., 2017; Lambert, Léonard, Drion, et al., 2013; Lambert, Léonard, Lecloux, et al., 2013; Omori et al., 2018; Scala et al., 2015). The results reported from histological analyses performed on the same material used in the present article represented the healing evaluated in two histological slides that were taken from the center of the experimental region (Iida et al., 2017). This histological analysis allowed the recognition of the various tissues involved in this region. However, larger volumes of the augmented sinus were not studied. Performing a micro‐computed tomography (CT) instead allowed an expansion of the analyses in a larger volume of the elevated spaces in 3D using hundreds of slides for analysis. Different thresholds of grays have been applied to identify new bone tissue and biomaterials used for ridge preservation (De Barros, Novaes, de Carvalho, & de Almeida, 2017), sinus elevation (Lim, Zhang, Lee, Jung, & Choi, 2015), or rat calvarial defects (Park et al., 2011). Moreover, it has been suggested that a low radio‐opacity threshold could include in the analysis immature bone tissue, especially in the earliest periods of healing (Park et al., 2011). Guidelines for terminology, technical procedures, and minimal set of variables that should be reported in a micro‐CT analysis have been illustrated (Bouxsein et al., 2010). However, a comparison between histological and micro‐CT data using different thresholds of grays when a xenograft is used may be helpful to understand better the interpretation of the data provided by a micro‐CT.

Hence, the aim of the present study was to evaluate possible differences in the assessment of bone formation between histological and micro‐CT analyses in maxillary sinuses augmented with a xenograft with similar density and mineral content of bone.

## 2 MATERIAL AND METHODS

The local ethics committee of the Faculty of Dentistry of Ribeirão Preto of São Paulo University, Brazil, approved the protocol before starting the experiment (2015.1.834.58.7). All procedures were carried out matching all regulations for animal care in Brazil. The recommendations suggested by ARRIVE were followed.

In a previous report, the histomorphometric data of the sinuses of test and control sites were presented (Iida et al., 2017). For detailed information, the reader is referred to that article. In the present paper, comparisons between test and control sites were not considered, whereas comparisons between histological and micro‐CT data represented the topic of interest.

Eighteen male New Zealand white rabbits, 3.5–4.0 kg of weight and 5–6 months old, were used. The animals were randomly divided into three groups, euthanized after 2, 4, and 8 weeks, respectively.

### 2.1 Randomization and allocation concealment

Test and control sites were randomized electronically (randomization.com) by an author not involved in surgical procedures and sacrifices (D. B.). The information about the allocation was provided by another person not involved in the surgical procedure (S. P. X.) after the opening of the access window bilaterally.

### 2.2 Surgical procedures

The surgeries were carried out by a maxillofacial surgeon with long clinical experience (Iida et al., 2017). General anesthesia was performed with acepromazine subcutaneously (1.0 mg/kg) and a mix of xylazine (3.0 mg/kg) and 60‐mg/kg ketamine (50.0 mg/kg) im. Local anesthesia was finally administered.

For further details, the reader is referred to the report on histological data (Iida et al., 2017). In brief, an incision was performed following the exposure of the nasal bone, antrostomies were performed bilaterally, and the sinus mucosa was elevated. Only at the test sites, an equine collagen membrane (OsteoBiol® Evolution 0.3 mm, Tecnoss®, Giaveno, Italy) was placed subjacent the sinus mucosa. Both sinuses were subsequently filled with similar amounts of collagenated cortico‐cancellous porcine bone (OsteoBiol Gen‐Os®, Tecnoss®, Giaveno, Italy; 250–1,000 μm). A small screw was placed as reference, and both antrostomies were covered with collagen membranes similar to the previous ones. The flaps were sutured, and antibiotic and analgesic/anti‐inflammatory drugs were administrated intramuscular.

### 2.3 Maintenance care

The animals were maintained at the university animal facilities in individual cages and in acclimatized rooms. The continuing monitoring of the postoperative animal conditions, feeding, and excretion were carried out.

### 2.4 Euthanasia

The euthanasia was carried out using an overdose of sodium thiopental (1.0 g, 2 ml, Thiopentax) administered intravenously. The biopsies obtained were representing the healing after 2, 4, and 8 weeks (*n* = 6 per observation period).

### 2.5 Micro‐CT evaluations

After the fixation in 10% buffered formalin, the small reference screws were removed for the micro‐CT exam and replaced in situ afterwards for the histological procedures. The biopsies were scanned in a high‐resolution micro‐CT 1172 (Bruker, Kontich, Belgium) using a resolution of 9.92‐μm isotropic pixel at 60 kV/165 μA and a filter Al 0.5 mm. The exposure was 596 ms, the rotation step amounted to 0.4°, the frame averaged was 4, and the random movement was 10. The micro‐CT cross‐sectional images were repositioned using the software DataViewer® (Bruker, Kontich, Belgium). Subsequently, basic and additional parameters were evaluated using the software CTan (Bruker, Kontich, Belgium).

**Figure 1:**
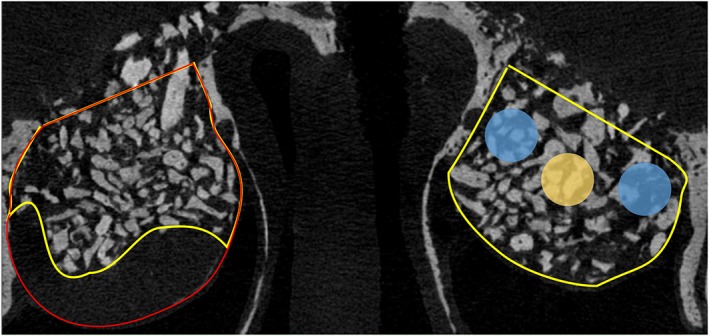
Drawing exemplifying the volumes and regions analyzed. The volume included in the analyses of the augmented sinus is outlined in yellow. The volume occupied by the inner collagen membrane was excluded from the analyses (outlined in red). Three interpolated cylindrical regions of 1 mm in diameter were used, located close to the medial and lateral bony walls (Walls region; light blue circle) and in the middle of the augmented volume (Middle region; yellow circle)

At the test site, the space occupied by the collagen membrane subjacent the sinus mucosa was excluded from the analyses. In an anterior–posterior plane, a region of 2 mm anteriorly or posteriorly (4 mm in total) to the center of the osteotomy was evaluated. Moreover, three interpolated cylindrical regions of 1 mm in diameter were also evaluated (Figure 1). Two of these regions were placed close to the medial and lateral bony walls, and mean values were calculated (*Walls* region). The other region (*Middle* region) was located in the middle of the elevated space.

The thresholds of gray levels to identify bone tissue and xenograft were set at 60–120 and 120–160, respectively. However, with an explorative aim, thresholds of gray of 80–120 and 120–180, for bone and xenograft, respectively, were used to evaluate possible variations compared with the previous basic set of thresholds. Subsequently, the cross sections were interpolated to obtain all volumes of interests.

### 2.6 Histological preparation and analyses

Following the micro‐CT analyses, two ground sections of each sinus were prepared and stained with either toluidine blue or Stevenel's blue and alizarin red (for details, see Iida et al., 2017).

A point counting procedure (Schroeder & Münzel‐Pedrazzoli, 1973) was used to evaluate the proportions of new bone and xenograft within the sinuses. Data from regions close to the medial and lateral bone walls of the sinus (Walls region) and from the center of the sinus (Middle region) were also analyzed separately.

### 2.7 Data analyses

Mean values between test and control were calculated for each animal for both micro‐CT and histology. The primary variable was mineralized bone percentage, whereas the secondary variable was the xenograft percentage.

The Wilcoxon rank sum test using the software IBM SPSS Statistics (IBM Inc., Chicago, IL, USA) was applied to evaluate differences between histological and micro‐CT data for mineralized bone and xenograft. The level of significance was set at 5%.

## 3 RESULTS

The data from a rabbit belonging to the 4‐week period were missing at the time of the analysis, and this animal was excluded from further analysis so that an *n* = 6 was reached for the 2‐ and 8‐week periods of healing, whereas for the 4‐week period, an *n* = 5 was obtained.

### 3.1 Volumetric evaluations

**Figure 2:**
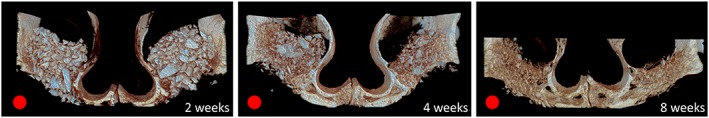
Three‐dimensional images of test (red circle) and control sites after 2, 4, and 8 weeks

The volumes of the *augmented space* decreased over time (Figure 2). Between 2 and 8 weeks of healing, the volume at the sites with the inner collagen membrane decreased from 30.0 to 23.4 mm^3^ (21.9% of reduction) whereas at the sites without the collagen membrane, the volume decreased from 36.2 to 26.3 mm^3^ (27.3% of reduction). The volumes evaluated in the Walls and Middle regions were about 3.1–3.2 mm^3^.

### 3.2 Morphometric evaluations in the augmented sinus (Table 1, Figure 3)

**Table 1:** Percentages of mineralized bone and xenograft evaluated within the augmented sinus in the various periods of healing

	Mineralized bone			Xenograft		
	Histo	Micro‐CT 60–120	Micro‐CT 80–120	Histo	Micro‐CT 120–160	Micro‐CT 120–180
2 weeks	7.5 ± 2.4	18.2 ± 1.7*	11.6 ± 1.2*	32.4 ± 3.5	18.2 ± 1.7*	21.3 ± 1.8*
4 weeks	15.7 ± 12.1	18.1 ± 4.0	13.1 ± 5.7	17.7 ± 6.1	11.9 ± 3.1	14.4 ± 3.4
8 weeks	27.0 ± 5.3	23.7 ± 6.4*	16.7 ± 4.9*	8.7 ± 4.3	6.9 ± 0.8	7.2 ± 0.9

*Note*. The micro‐computed tomography (CT) analyses were performed using two different level of grays.

* *P* < 0.05 between the histological data and those from the micro‐CT analyses.

**Figure 3:**
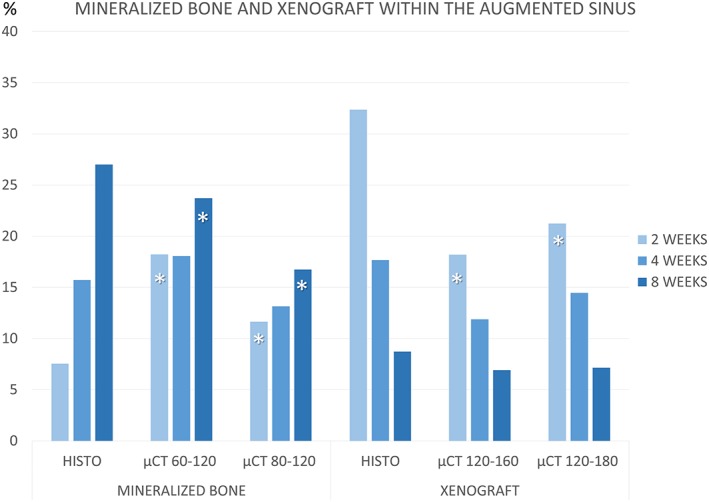
Graphs comparing percentages of new mineralized bone and xenograft evaluated within the augmented sinuses at different periods of healing, using thresholds set with gray levels of 60–120 and 120–160 or 80–120 and 120–180, for bone and xenograft, respectively

At the histological analyses, bone increased over time from 7.5 ± 2.4% to 27.0 ± 5.3% between 2 and 8 weeks of healing. A similar trend of bone formation was shown at the micro‐CT analyses, for both 60–120 and 80–120 levels of grays, however presenting higher proportions after 2 weeks and lower proportions after 8 weeks of healing compared with the histological analyses. The differences between the histological and micro‐CT data were statistically significant for both periods, whereas the correlation was weak positive after 2 weeks and strong positive after 8 weeks of healing for both level of grays.

The percentages of xenograft decreased over time, showing similar trend at the histological and micro‐CT analyses. However, at the 2‐week period, the percentages encountered at the histological analyses were higher compared with those measured at the micro‐CT, being both differences statistically significant. However, after 8 weeks of healing, similar percentages of xenograft were detected in both analyses.

### 3.3 Morphometric evaluations in the Bone walls and Middle regions (Table 2, Figure 4)

**Table 2:** Percentages of mineralized bone and xenograft evaluated in the Bone walls and Middle regions in the various periods of healing

	Mineralized bone				Xenograft		
	Region	Histo	Micro‐CT 60–120	Micro‐CT 80–120	Histo	Micro‐CT 120–160	Micro‐CT 120–180
2 weeks	Bone walls	11.7 ± 3.7	18.1 ± 2.0*	11.6 ± 1.3	25.3 ± 4.6	17.2 ± 2.6*	20.0 ± 3.3
	Middle	1.6 ± 1.7	22.9 ± 2.6*	15.0 ± 1.9*	41.5 ± 9.6	22.3 ± 5.3*	26.5 ± 2.6*
4 weeks	Bone walls	21.9 ± 17.7	20.0 ± 4.5	12.2 ± 3.2	13.0 ± 5.6	11.1 ± 3.5	14.3 ± 5.3
	Middle	8.2 ± 9.7	15.5 ± 2.7	9.9 ± 1.9	18.5 ± 10.3	15.4 ± 5.6	21.1 ± 7.9
8 weeks	Bone walls	32.1 ± 5.1	26.0 ± 5.0*	18.5 ± 4.0*	6.9 ± 2.0	9.1 ± 3.2	8.8 ± 2.5
	Middle	18.5 ± 13.5	21.7 ± 9.6	14.9 ± 7.5	11.0 ± 6.9	5.6 ± 1.9	6.2 ± 2.2

*Note*. The micro‐computed tomography (CT) analyses were performed using two different level of grays.

* *P* < 0.05 between the histological data and those from the micro‐CT analyses.

**Figure 4:**
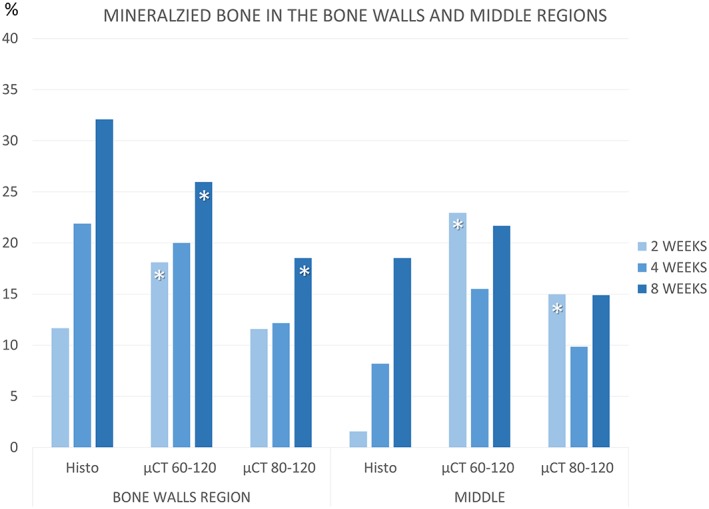
Graphs comparing percentages of new mineralized bone evaluated in the Bone walls and Middle regions at different periods of healing, using thresholds set with gray levels of 60–120 and 80–120

In the *Bone walls* region, bone was found increasing between 2 and 8 weeks of healing at both analyses. After 2 weeks of healing, the percentage of new bone at the histological analyses was similar to that detected at the 80–120 level of grays, whereas it was lower compared with that evaluated using a threshold of 60–120, being the differences statistically significant. The correlation with the histological data was moderate positive for both gray levels.

After 8 weeks of healing, higher percentages of bone were found at the histological compared with the micro‐CT analyses, being the differences statistically significant. The correlation was weak positive for both thresholds.

In the Middle region, at the histological analyses, very little bone was found (1.6 ± 1.7%) whereas at the micro‐CT analyses, statistically significant higher percentages of bone were found for both threshold of 60–120 (22.9 ± 2.6%) and 80–120 (15.0 ± 1.9%) levels of gray. A weak negative correlation was found between histological and micro‐CT data, for both levels of grays used. At the histological analyses, new bone was found increasing over time. At the micro‐CT analyses, bone decreased after 4 weeks of healing and increased after 8 weeks, however, without reaching similar values found at the 2‐week period of evaluation. After 8 weeks, the correlation between histological data and micro‐CT analyses was strong positive for both levels of grays.

Similar trends of xenograft resorption were found at both analyses. After 2 weeks, higher content of xenograft was found at the histological compared with the micro‐CT analyses, especially in the Middle zone. However, similar percentages of residues of xenograft were found after 8 weeks of healing.

## 4 DISCUSSION

The aim of the present study was to evaluate possible differences in the assessment of bone formation between histological and micro‐CT analyses in maxillary sinuses augmented with a xenograft with similar density and mineral content of bone.

When a xenograft with similar characteristics of those of bone is used for sinus floor elevation, technical problems may occur in detecting the correct proportions of newly formed bone and residual biomaterial in the micro‐CT analysis. Moreover, the gray thresholds used for analysis strongly influenced the results.

At the histological analyses, low amount of new bone was seen after 2 weeks of healing in the grafted space (7.5%). This new bone was mainly localized towards the bone walls (11.7%) compared with the middle parts of the sinus (1.6%). In the next two periods of evaluation, bone increased in percentages reaching 27% as mean within the grafted sinus. However, still higher percentages of new bone were seen towards the Bone walls compared with the Middle region. These results are in agreement with other studies that showed that bone was formed from the preexisting bone of floor and walls of the sinus (Caneva et al., 2017; Favero et al., 2016; Omori et al., 2018; Scala et al., 2010, 2012, 2015, 2016).

In an experiment in rabbits in which a similar augmentation xenograft was used for sinus augmentation, after 2 weeks of healing, 3–4% of new bone was found close to the Bone walls whereas in the Middle zone, no new bone was detected (Omori et al., 2018). Bone increased to 10–11% close to the Bone walls and to 1.2–5.0% in the Middle region. After 8 weeks, similar amount of new bone was found at both regions.

In another similar experiment in rabbits, deproteinized bovine bone mineral was used and tissues evaluation was performed at various periods of healing. New bone was found at percentages of about 13%, 27%, 31%, and 36% close to the Bone walls, whereas in the Middle region, the percentages were about 0%, 6%, 12%, and 28%, after 7, 14, 21 and 40 days of healing, respectively.

Several studies have shown that the sinus mucosa, despite its potential of forming bone (Gruber, Kandler, Fuerst, Fisher, & Watzek, 2004; Srouji et al., 2009), did not effectively participate in bone formation in an “in vivo” environment. (Caneva et al., 2017; Favero et al., 2016; Omori et al., 2018; Scala et al., 2010, 2012, 2015, 2016). This, in turn, means that the contribution in bone formation within the middle regions depends predominantly on bone formation from the walls of the sinus and will occur subsequently compared with the regions closer to the walls.

After 2 weeks of healing, the evaluations performed at the micro‐CT within the augmented sinus revealed significant higher contents of new bone compared with the histological analyses. The difference was higher when the Middle zone was analyzed. This is in disagreement with the histological data from the present study and from the data of other studies above reported. Moreover, within the middle regions, higher amount of bone was found after 2 weeks compared with that after 4 weeks of healing in both thresholds of gray levels used.

Several factors may provide reasonable explanations of these incongruences in the outcomes between histological and micro‐CT assessments when a resorbable filler material similar to that of the present experiment was used.

In an in vitro study (Figueiredo et al., 2010), several parameters were evaluated, among which the density and the mineral content of different biomaterial. Density and mineral content of Gen‐Os® and natural human bone resulted to be very similar (2.43 g/cm^3^ and 64.6% for Gen‐Os® and 2.30 g/cm^3^ and 65.0% for human bone). Other biomaterials analyzed showed 2.92–3.21 g/cm^3^ and ≥95% of density and mineral content, respectively. This similarity between Gen‐Os® and bone may have engendered in the present study an overestimation in the micro‐CT analysis of the content of new bone when such biomaterial was present.

Another important factor that influenced the outcome in the micro‐CT analysis was the threshold of the gray levels used. Setting the lower parameter for gray level threshold to 80 instead of 60 resulted in a decreased volume and percentages of bone. This outcome is in agreement with a study in rats (Park et al., 2011), in which the correlation between histomorphometric analysis and micro‐CT analysis was studied in regenerated critical‐size calvarial defects. The percent of bone in the defect site was measured setting the lower threshold at 50, 60, 65, 70, and 80 of the greyscale index. Likewise to the present study, new bone volume decreased when the lower gray threshold level was enhanced. It was supposed that a lower level of threshold, such as 50, might include immature bone with low radiodensity in the early period of healing (after 2 weeks). However, after 4 weeks, bone was mature enough to be detected in the radiographic assessment (Park et al., 2011).

**Figure 5:**
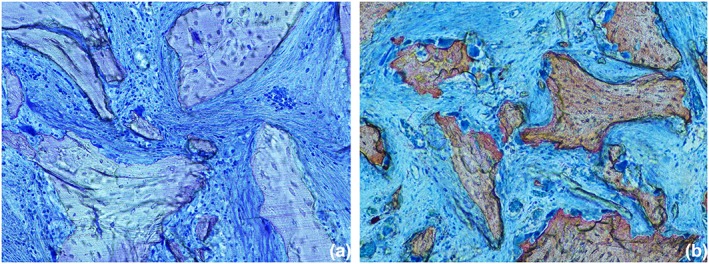
Microphotography of a ground section representing the healing after 2 weeks. (a) A dense tissue, rich in fibroblast‐like cells and fibers, was seen around the xenograft particles. Original magnification ×200. Toluidine blue stain. (b) Various osteoclasts cells located around the xenograft particles. Original magnification ×100. Stevenel's blue and alizarin red stain

**Figure 6:**
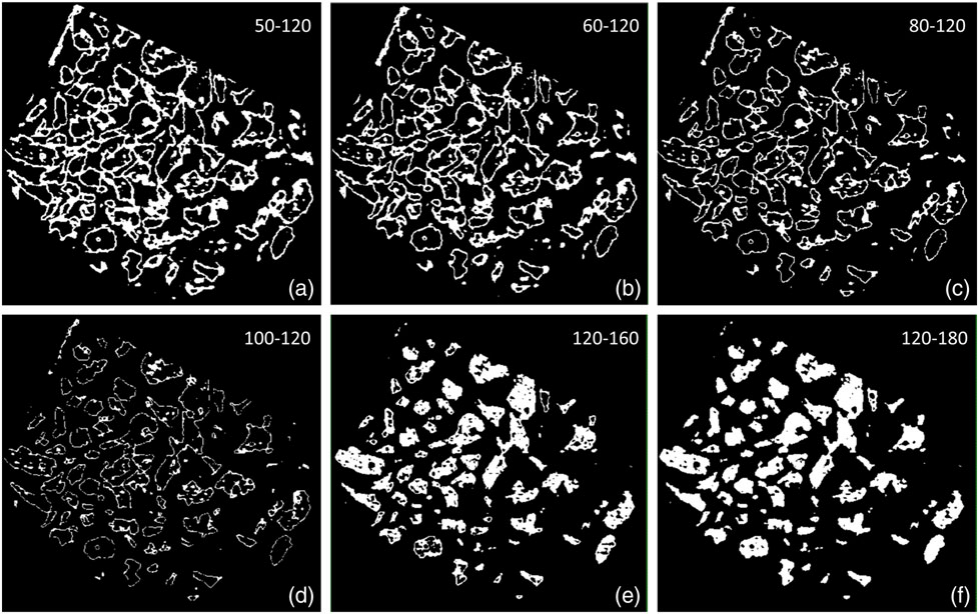
Binary section of a control volume of interest after 2 weeks of healing using different gray threshold levels: (a–d) 50–120, 60–120, 80–120, and 100–120 for bone detection and (e–f) 120–160 and 120–180 for xenograft detection. (a–d) Increasing progressively the lower level of gray thresholds from 50 to 100, a progressive decrease of the dimensions of the tissue surrounding the graft particles was detected. (e–f) Increasing the higher level of the gray thresholds, a higher density of xenograft was detected

In the present experiment, the higher values of newly formed bone obtained in the radiographic assessment compared with the histologic evaluation after 2 weeks of healing may also be attributed to the presence of osteoid tissue around the graft particles. In fact, in the histological analyses described in the previous report on the present experiment (Iida et al., 2017), a dense tissue, rich in fibroblast‐like cells and fibers, was seen around the particles (Figure 5a). Increasing progressively the lower level of gray thresholds from 50 to 120, a progressive decrease of the dimensions of this tissue surrounding the graft particles was seen (Figure 6a–f). A low threshold of gray might incorporate also this soft dense tissue around the particles. After 8 weeks, when the graft particles were reduced in percentage, this phenomenon was less evident. A similar dense tissue was also described around another type of xenograft particles (Caneva et al., 2017) as well as around implants installed in sites presenting marginal bone defects (Botticelli, Berglundh, Buser, & Lindhe, 2003; Rossi et al., 2014). It was suggested that this dense tissue might be regarded as an osteoid tissue (Botticelli et al., 2003).

Another factor that may have created false positive outcomes could be related to the resorption of the biomaterial. The osteoclastic activity resulted in the release of minerals within the surrounding soft tissue (Figure 5b; Shilling et al., 2006). It may be argued that this may result in an increase in density of the surrounding tissue that was detected by a lower level of threshold of grays.

The limitations of the present study are represented by the limited number of animals per periods and the animal model used so that any inference with the humans should be taken with caution.

In conclusion, the outcome of a micro‐CT analysis performed in an early phase of healing may be altered when a resorbable bone substitute with similar density and mineral content of bone is applied.

## CONFLICT OF INTERESTS

Dr. Daniele Botticelli declares to be co‐owner of Ariminum Odontologica and to be the principal investigator of ARDEC Academy. All the other authors declare no conflict of interest regarding this study.
